# Transcriptional response of *Lactococcus lactis* during bacterial emulsification

**DOI:** 10.1371/journal.pone.0220048

**Published:** 2019-07-25

**Authors:** Mariya Tarazanova, Thom Huppertz, Marjo Starrenburg, Tilman Todt, Sacha van Hijum, Jan Kok, Herwig Bachmann

**Affiliations:** 1 TI Food and Nutrition, AN Wageningen, The Netherlands; 2 NIZO, Ede BA, The Netherlands; 3 Molecular Genetics, University of Groningen, Groningen, The Netherlands; 4 Radboud University Medical Centre CMBI, Geert Grooteplein Nijmegen, The Netherlands; 5 HAN, University of Applied Sciences, PGL Nijmegen, The Netherlands; LAAS-CNRS, FRANCE

## Abstract

Microbial surface properties are important for interactions with the environment in which cells reside. Surface properties of lactic acid bacteria significantly vary and some strains can form strong emulsions when mixed with a hydrocarbon. *Lactococcus lactis* NCDO712 forms oil-in-water emulsions upon mixing of a cell suspension with petroleum. In the emulsion the bacteria locate at the oil-water interphase which is consistent with Pickering stabilization. Cells of strain NCDO712 mixed with sunflower seed oil did not stabilize the oil droplets. This study shows that the addition of either ethanol or ammonium sulfate led to cell aggregation, which subsequently allowed stabilizing oil-in-water emulsions. From this, we conclude that bacterial cell aggregation is important for emulsion droplet stabilization. To determine how bacterial emulsification influences the microbial transcriptome RNAseq analysis was performed on lactococci taken from the oil-water interphase. In comparison to cells in suspension 72 genes were significantly differentially expressed with a more than 4-fold difference. The majority of these genes encode proteins involved in transport processes and the metabolism of amino acids, carbohydrates and ions. Especially the proportion of genes belonging to the CodY regulon was high. Our results also point out that in a complex environment such as food fermentations a heterogeneous response of microbes might be caused by microbe-matrix interactions. In addition, microdroplet technologies are increasingly used in research. The understanding of interactions between bacterial cells and oil-water interphases is of importance for conducting and interpreting such experiments.

## Introduction

The interactions between microbial cells and substrates or solid surfaces can be attractive or repulsive and depend on properties such as temperature, pH, ionic strength, roughness of a surface, hydrophobicity or surface charges [1,2]. Bacterial adhesion has been studied in relation to bacterial infections [3], adhesion to environmental systems, e.g., intertidal systems with subsequent biofilm formation [4–6], biomedical applications [7], as well as bioremediation and fermentation processes [8–10]. Gram-positive bacteria have been shown to function as emulsifiers of hydrocarbons e.g., petroleum, without involvement of cell growth and substrate degradation [11–13]. Such emulsification is caused by microbial cells locating on the oil-water interphase, which prevents droplet coalescence and leads to so called Pickering-stabilization of emulsions [14,15]. The droplet size distribution of emulsions stabilized by microbial cells is in the range between 100–500 μm [16]; the stabilizing particle size should be at least an order of magnitude smaller than the emulsion droplet size [17]. Bacterial cells are often simplified to solid particles in order to describe such emulsions [18,19]. The contacts between these solid particles and the surfaces of emulsion droplets are typically explained by van der Waals and electrostatic interactions and they are united in the so-called DLVO theory (named after Derjaguin, Landau, Verwey, and Overbeek) [20]. Most bacterial surfaces are negatively charged and can be regarded as charged colloidal particles in aqueous systems [16,19]. Bacterial cell wall molecules such as proteins or polysaccharides will attract counter ions from the surrounding environment and, together, form an electrical double layer around the cell [21]. Thus, the pH and ion concentration of the surrounding environment has been suggested to affect the location of bacteria at the oil-water interphase of an emulsion [22]. However, a generic explanation for microbe-matrix adhesion interactions was not obtained by considering bacteria as charged colloidal particles with a surrounding electric double layer. The addition of short-range Lewis acid-base interactions or hydration, and steric interactions led to the extended XDLVO theory [19,23]. However, even XDLVO does not fully explain microbe-matrix interactions, probably because of the high cell surface complexity, which significantly differs between bacteria and non-biological particles [18,24]. Indeed, the molecular composition of Gram-positive bacterial cell surfaces is quite diverse [25,26], providing cells with different surface properties [27–29]. The resulting differences in e.g. charge [25,30] and hydrophobicity [31,32] are involved in bacterial interactions with interphases [2,33]. By contrast, the surface of solid spherical particles is uniformly charged or hydrophobic. Adding to the complexity is the fact that interactions between bacteria and substrates can be strain-specific [15,34].

Examples of undesirable bacterial emulsification can be found in biofuel production where oil-producing bacteria can stabilize biofuel oil droplets as Pickering stabilization particles in water, which further impedes biofuel recovery [35]. The number of bacterial species that have been described to facilitate Pickering stabilization is still limited [12,16,36]. It was recently reported that lactic acid bacteria (LAB) can be applied as solid particles for the production of food-grade Pickering emulsions [16,37]. The influence of LAB surface properties on food emulsions already stabilized by a surfactant was investigated and the results suggest that they play an important role in interactions of bacteria with emulsion matrix components [38,39]. We recently also showed that by altering the surface of the LAB *Lactococcus lactis* through e.g. the overexpression of lactococcal pili, the gel hardness and the viscosity of a fermented milk product made with this organism were changed [40]. While there is a reasonable amount of knowledge on how bacteria in food fermentations influence textural properties of the fermented food matrix [10,41–43], little information is available on molecular mechanism involved in these interactions [27,41] or on whether and to which extent the location of bacteria on e.g. an oil-water interphase might influence their behaviour.

We hypothesized that altering cell surface properties may allow changing emulsification properties of bacteria. This supposition is based on the fact that chemicals such as acetic and succinic anhydrides, carbodiimide and ethanolamine or ethylenediamine can modify cell surface charge, isoelectric point or water contact angles [44]. Calcium ions influence bacterial adhesion to piglet epithelial cells [45], high concentrations of ammonium sulfate cause cell aggregation [46] and even small differences in growth media can change the bacterial cell surface properties [47]. Here we used *L*. *lactis* to prepare Pickering emulsions with either petroleum, sunflower seed oil or the fluorinated oil HFE7500. We show that cell aggregation caused by ammonium sulfate or ethanol influences bacterial emulsification of sunflower seed oil. Furthermore we investigated how bacterial emulsification through Pickering stabilization of HFE7500 influences the transcriptional response of the cells.

## Results

### *Lactococcus lactis* can stabilize oil-in-water emulsions

**Fig 1:**
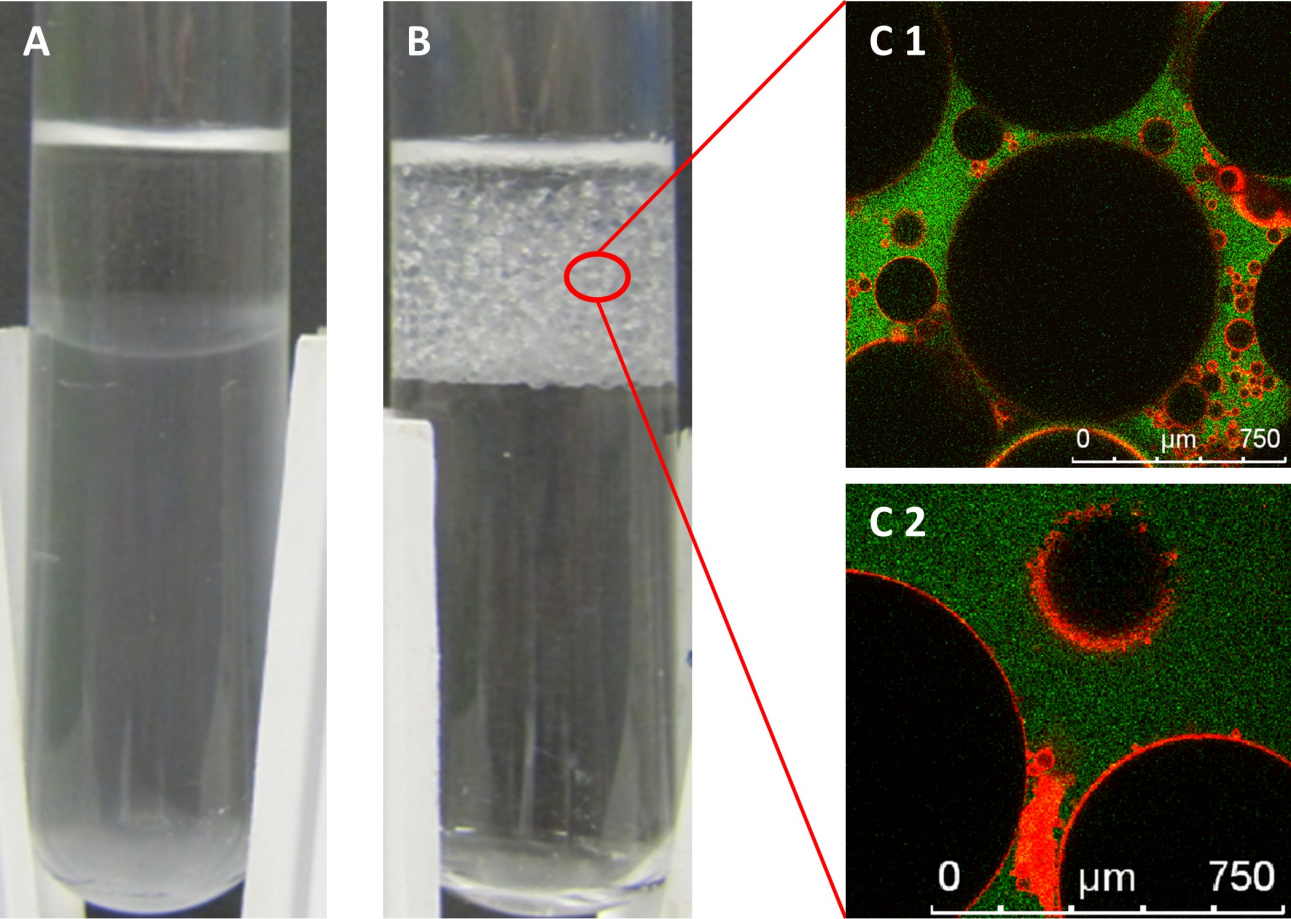
Emulsification of petroleum by *L*. *lactis*. (A) A suspension of overnight-grown *L*. *lactis* MG1363 cells in 10 mM phosphate buffer (pH 6.8), after vigorous shaking with petroleum, shows no emulsification of the oil phase (top layer). The cells can be seen in the lower phase (compare with (B)). (B) *L*. *lactis* NCDO712 produces an emulsion in petroleum with 99% of the cells residing at the oil-water interphase (top layer). (C) CLSM image of the oil-in-water emulsion made with *L*. *lactis* NCDO712. Petroleum droplets are not fluorescent (black), buffer containing the dye carboxyfluorescein is green (continuous phase) and bacterial cells are red. Due to the polydispersity of the droplets the position in depth differs for individual droplets and therefore different densities of cells are visualized on the oil-water interphase. Size marker is indicated in white.

In an earlier characterization of *L*. *lactis* cell surface properties we found considerable diversity between the propensity of strains to emulsify hydrocarbons but no correlation between emulsion stabilization and cell surface hydrophobicity was found [29]. For the further investigation two strains with the same genetic background but with opposite emulsification properties were selected (Table A in S1 Tables). *L*. *lactis* NCDO712 cells (99% hydrophobicity) form emulsions when they are mixed with petroleum (Fig 1B) while cells of *L*. *lactis* MG1363, a plasmid-free derivative of strain NCDO712, (6% hydrophobicity) do not form such emulsions (Fig 1A). Interestingly, the overexpression of the lactococcal pilin gene cluster *pil*, in strain MG1363*pil*, lead to high cell surface hydrophobicity and strong emulsification properties when mixed with petroleum [40]. To identify the type of emulsion formed by strain NCDO712 we labelled the water phase (buffer) with the green fluorescent dye carboxyfluorescein and the bacterial cells with the DNA stain Syto 60, which fluoresces in the red spectrum. Subsequently, we analysed the emulsion using confocal laser scanning microscopy (CLSM). The images show that a dense layer of bacterial cells surrounds the petroleum droplets while the buffer forms the continuous phase of the emulsion (Fig 1C and S1 and S2 Movies). This analysis established that the bacterial cells are located at the oil-water interphase, forming an oil-in-water Pickering emulsion (Fig 1C).

### Cell aggregation influences cell emulsification properties

**Fig 2:**
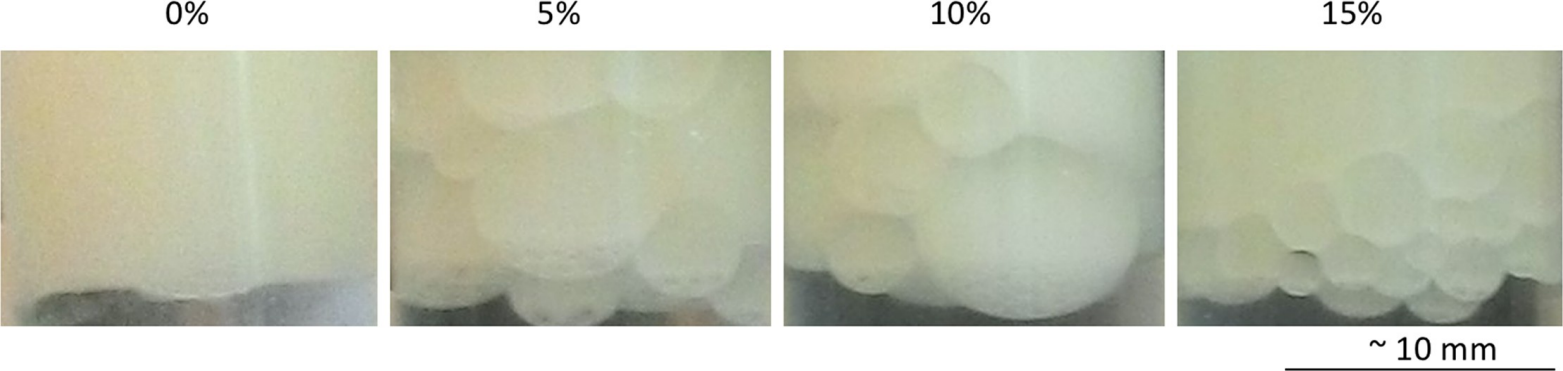
Emulsification of sunflower seed oil as a result of *L*. *lactis* NCOD712 cell aggregation upon ethanol addition. Addition of ethanol (% (v/v), percentages given above the pictures) to 5 ml of cells from a stationary phase culture of *L*. *lactis* NCOD712 (OD_600_ = 1), re-suspended in 10 mM phosphate buffer (pH 6.8) allows stabilizing sunflower seed oil emulsion droplets. Ethanol concentrations higher than 20% did not lead to considerable emulsification. Controls without cells did not lead to emulsion droplet stabilization.

Pickering emulsification of petroleum was easily done with strain NCDO712, however, with sunflower seed oil, which was free from natural emulsifiers, no or only little emulsification was observed (Fig 2).

The emulsification results suggested that strains with a clumping/aggregating phenotype are more likely to emulsify petroleum as NCDO712, MG1363*pil* and MG1614_clu^+^ do (Table A in S1 Tables), compared to strains that do not aggregate [29]. Based on this observation we hypothesized that cell aggregation might contribute to bacterial emulsification, which is supported by the fact that the energy needed for the detachment of particles from an interphase in a Pickering emulsion increases with the radius of the particles [17,48]. Ammonium sulfate can cause cell aggregation by a mechanism of “salting out” of proteins [46]. We also tested whether ethanol could aggregate cells and found that the addition of both, AMS or ethanol leads to the formation of cell aggregates (Table 1 and S1 Fig). Next we tested to what extent induced cell aggregation would influence emulsification. Control samples without cells, consisting of buffer with ethanol or ammonium sulfate and sunflower seed oil only, did not result in any emulsion formation. Aggregation of cells in a buffer was observed 1 h after addition of more than 5% ethanol or 0.1–3.0 M ammonium sulfate. The addition of 5% ethanol to the cell suspension led to an increase in cell aggregation and the propensity to form emulsions with sunflower seed oil (Table 1, Fig 2). The further increase of the ethanol concentration gave variable results (CSH dropped to ~32% after addition of 80% ethanol while aggregation varied from 10–55% with ethanol concentrations above 25%) which might be due to effects of ethanol on the cell surface other than cell clumping. The fact that higher ethanol concentrations did not lead to more emulsification (increase in CSH) argues against the possibility that the release of cell content in response to ethanol might facilitate emulsification. The addition of 0.1 M ammonium sulfate led to a clear increase of surface hydrophobicity while concentrations of 2 M or more were needed to see measureable effects on cell aggregation (Table 1). The fact that either the addition of ethanol or ammonium sulfate resulted in increased cell aggregation and subsequently improved oil emulsification, suggests that cell aggregation aids bacterial emulsification properties.

**Table 1:** Cell surface hydrophobicity (CSH, %) of *L*. *lactis* NCDO712 in sunflower seed oil-based emulsions under cell aggregation conditions: Ethanol or ammonium sulfate. Results are the average (AV) of 3 replications with standard deviation (STD).

Ethanol			Ammonium sulfate		
Concentration, %	Aggregation, %	CSH, %	Concentration, M	Aggregation, %	CSH, %
	AV±STD	AV±STD		AV±STD	AV±STD
0	12.1 ± 0.1	0.1 ± 0.1	0	8.4 ± 0.9	0.1 ± 0.1
5	52 ± 0.6	74.7 ± 3.5	0.1	1.7 ± 1.5	73.6 ± 0.4
10	54.4 ± 0.9	66 ± 1.8	0.5	5.6 ± 0.6	44.6 ± 0.5
15	31.3 ± 1.1	67 ± 2.5	1.0	2.2 ± 1.2	55.6 ± 0.5
20	32.3 ± 1.7	87.6 ± 0.1	2.0	16.2 ± 1.1	96.2 ± 0.5
25	7 ± 6.1	50.1 ± 1.1	3.0	80.6 ± 4.4	99.7 ± 0.1

### Transcriptome response of *L*. *lactis* cells residing at an oil-water interphase

**Fig 3:**
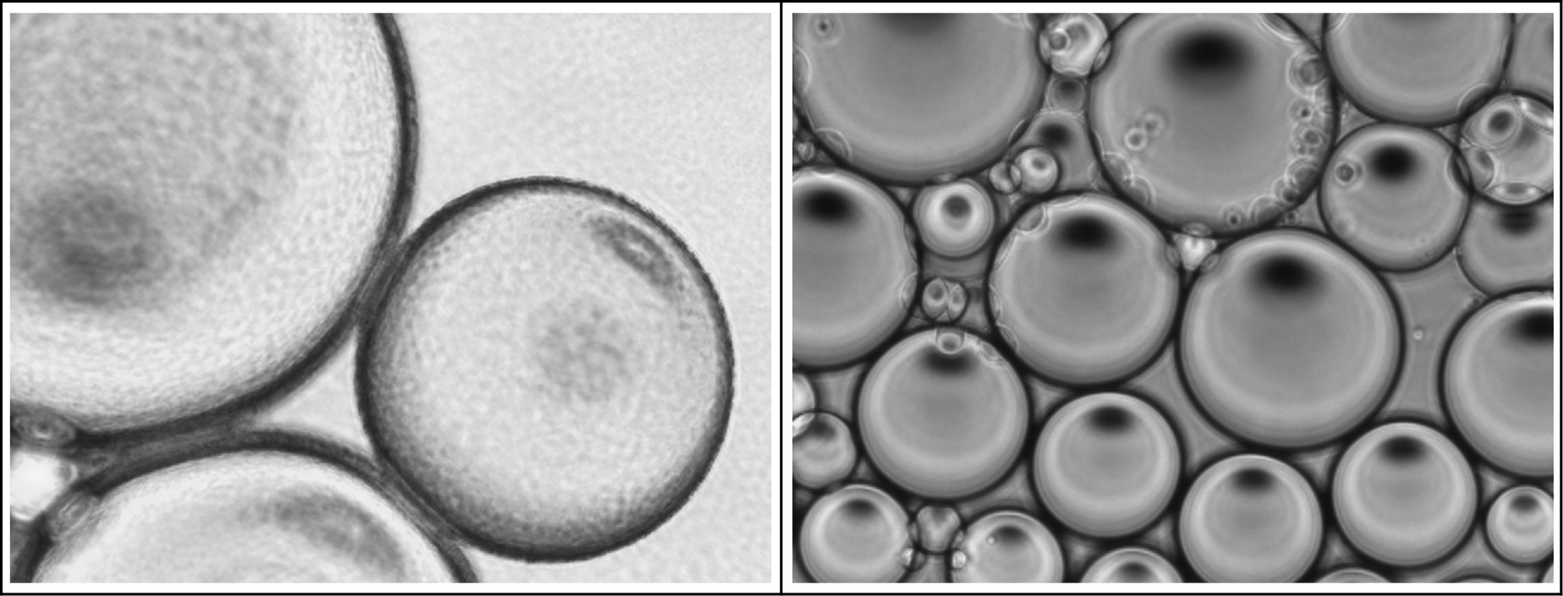
Oil-in-water emulsion stabilized by *L*. *lactis* NCDO712. Fluorinated oil (2 ml HFE7500) was mixed by vortexing with 5 ml of cell suspension (OD_600_ = 1) in 10 mM phosphate buffer (pH 6.8). The rough droplet surface is caused by multiple layers of bacterial cells covering the droplets (left panel). For comparison—the surface is smooth when water droplets are stabilized using the same oil but supplemented with a surfactant [49] (right panel).

While there is a rich body of knowledge on how starter culture cells can influence the properties of the food matrix during fermentation, little is known on possible converse interactions. To obtain more insights to which extent the product matrix influences the bacteria, we prepared Pickering-type emulsions with *L*. *lactis* NCDO712, were the cells are located on the oil-water interphase and the transcriptome response was determined. For this *L*. *lactis* NCDO712 cells were taken either from a suspension or from the oil-water interphase of an emulsion 0, 10, 20 or 30 min after emulsion preparation. RNA was subsequently isolated for RNAseq analysis. Emulsions were made with the fluorinated oil HFE7500 (Fig 3), which is considered nontoxic as it allows culturing of lactococci in water-in-oil emulsions prepared with it [49]. The majority of cells in such a system are located on the oil-water interphase, which can be deduced from the fact that an increase in the number of cells added to the system allows to generate larger numbers of smaller oil droplets. To get an indication that *L*. *lactis* survives on an oil water interphase with HFE7500 we determined colony forming units (CFUs) 30 minutes after emulsion preparation and compared it to the cell suspension used to prepare the emulsion. While a direct comparison of emulsions with suspensions is difficult due to high cell concentrations around oil droplets but low cell concentrations in the water phase next to it both samples showed similar cell densities of approximately 1e10 cells/ml after the incubation.

**Fig 4:**
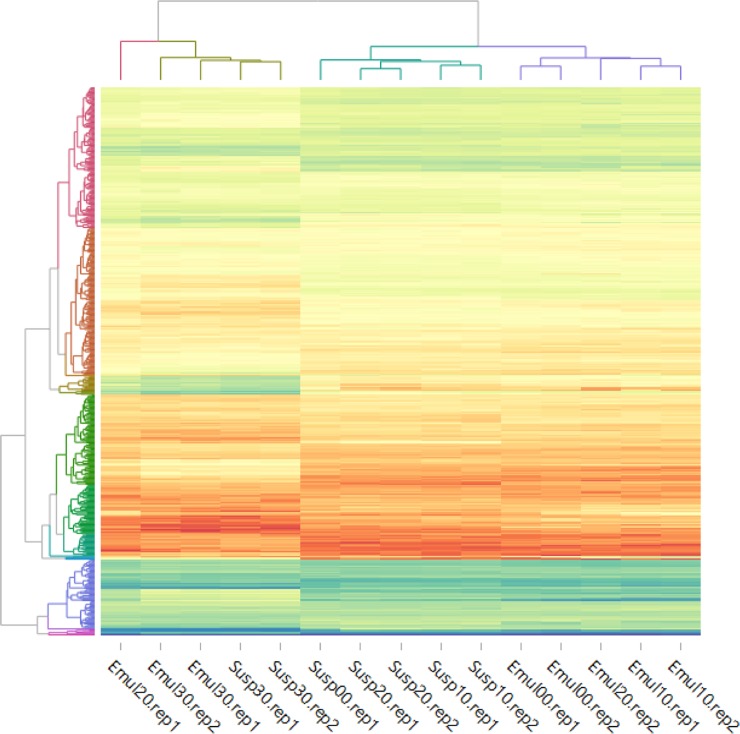
Clustering of RNAseq data based on normalized counts of all expressed genes in *L*. *lactis* NCDO712. Conditions are labelled by culture medium (emulsion–Emul, suspension–Susp), time of incubation (00–0 min, 10–10 min, 20–20 min, 30–30 min), and biological replication (biological replication 1 or 2—rep1 or rep2). The data of the biological replication 2, cells incubated in suspension for 0 min (Susp00.rep2) was omitted from the analysis due to poor quality of the sample.

The RNAseq data showed that replicate samples cluster together as expected. Clustering was also observed for either emulsion or suspension samples taken at 0, 10 and 20 min (Fig 4). The transcriptional response of lactococcal cells to residing at an oil-water interphase differs from that of cells in suspension at the equivalent time point. Interestingly, the transcriptomic response of the cells present for 30 min in emulsion converges to that in the cells kept for 30 min in suspension. Due to the high cell densities required to form a proper emulsion, it is likely that the transcriptional response after 30 min is dominated by acidification and subsequent entering into the stationary growth phase. One of the emulsion samples taken after 20 min of incubation, Emul20.rep1, clusters with the 30 min samples (Fig 4), suggesting that the cells in this sample reached stationary phase somewhat earlier.

For a more detailed analysis of gene expression under the two conditions employed, genes with higher than 4-fold differential expression (*p*-value < 0.01) were selected. The analysis was performed for all replicate samples taken after 10 min of incubation of the cells in both conditions because the initial transcriptional response of the lactococcal cells residing on an oil-water interphase differs from that of the cells in the suspension at the equivalent time point (10 min) according to cluster analysis. In total 72 genes were thus analysed, 28 of which are involved in amino acid transport and metabolism (Table 2 and Table B in S1 Tables). Other groups of highly differentially expressed genes encode proteins involved in inorganic ion transport and metabolism (10 genes) and sugar transport and metabolism (6 genes). Another 16 genes have unknown or no predicted functions (Table B in S1 Tables). In contrast to the 10 min samples, the 30 min samples converged, possibly because cells reached stationary phase, which resulted in only 2 genes being differentially expressed: *enoB* (*llmg_pseudo_08*) (7.0 fold change; p = 0.0007) and *pSH73_05* encoding a hypothetical protein (-18.4 fold change, p = 0.002).

**Table 2:** Differential gene expression in *L*. *lactis* NCDO712. Genes presented were more than 4-fold differentially expressed (*p*-value < 0.01) after 10 min of incubation in an oil-in-water emulsion compared to cells in suspension. The genes are classified according to their COG functions [50]. Gene clusters according to [51] are marked in bold.

Gene ID ^COG function^*	Gene Name	Gene annotation	Fold change of Emul10/Susp10	*p*-value
**C. Energy production and conversion**				
llmg_0635	*gltA*	citrate synthase	6.5	8.1e-4
llmg_0636	*citB*	aconitate hydratase	13.9	6.9e-8
llmg_0637	*icd*	isocitrate dehydrogenase	11.3	4.7e-9
**E. Amino acid transport and metabolism**				
llmg_0362	***dppA***	dipeptide-binding protein precursor	45.3	1.9e-7
llmg_pseudo_09	***dppP***	dipeptide-binding protein	4.6	1.1e-6
**llmg_pseudo_42**	*leuB*	isocitrate/isopropylmalate dehydrogenase	14.9	1.4e-13
**llmg_pseudo_43**	*leuA*	2-Isopropylmalate synthase	14.9	3.2e-9
**llmg_1284**	*leuC*	isopropylmalate isomerase large subunit	13	2.4e-12
llmg_0118	*ctrA*	branched chain amino-acid transporter (BcaP)	7.5	2.4e-6
llmg_pseudo_64	***oppF2***	oligopeptide transport ATP-binding protein	5.7	1.1e-6
llmg_pseudo_65	***oppD2***	oligopeptide transport ATP-binding protein	7.5	6.6e-7
llmg_2024	*oppA2*	oligopeptide-binding protein oppA2 precursor	4	2.4e-6
llmg_2026	*oppB2*	peptide transport system permease oppB2	5.3	2.1e-6
llmg_0697	***oppD***	oligopeptide transport ATP-binding protein oppD	5.3	4.2e-5
llmg_0698	***oppF***	oligopeptide transport ATP-binding protein oppF	5.6	5.2e-6
llmg_0699	***oppB***	peptide transport system permease oppB	5.3	2.2e-5
llmg_0700	***oppC***	oligopeptide transport system permease oppC	5.3	1.9e-5
llmg_0096	*llmg_0096*	glyoxylase	4.9	9.6e-7
llmg_1295	***hisD***	HisD protein	4.9 4	2.1e-6
llmg_1296	***hisG***	ATP phosphoribosyltransferase	4.6	1.2e-5
llmg_1297	***hisZ***	ATP phosphoribosyltransferase	6.9	1.2e-10
llmg_1298	***hisC***	histidinol-phosphate aminotransferase	8.6	1.2e-10
**llmg_1279**	*ilvB*	acetolactate synthase catalytic subunit	4.9	1.9e-7
**llmg_1280**	*ilvD*	dihydroxy-acid dehydratase	4.9	3.6e-8
llmg_1183	*gltB*	glutamate synthase. large subunit	4.3	3.4e-4
llmg_1290	***hisF***	imidazole glycerol phosphate synthase subunit HisF	4.9	1.9e-4
llmg_1291	***hisA***	1-(5-phosphoribosyl)-5-[(5- phosphoribosylamino)methylideneamino] imidazole-4-carboxamide isomerase	4.3	1.2e-4
llmg_1278	*ilvH*	acetolactate synthase 3 regulatory subunit	4	1.1e-5
llmg_1452	*llmg_1452*	amino-acid permease	-4	1.2e-4
llmg_1993	*llmg_1993*	transporter	-4.3	1.6e-7
**G. Carbohydrate transport and metabolism**				
llmg_1873	***glgD***	glucose-1-phosphate adenylyltransferase	8.6	7.2e-6
llmg_1874	***glgC***	glucose-1-phosphate adenylyltransferase	5.7	6.1e-4
**llmg_0966**^**K**^	*rmaI*	MarR family transcriptional regulator	5.3	4.1e-4
**llmg_0967**	*llmg_0967*	permease	8	2.9e-6
llmg_0022	*mtlA*	PTS system mannitol-specific transporter subunit IIBC	4.3	2.6e-4
**I. Lipid transport and metabolism** **Q. Secondary metabolites biosynthesis, transport, and catabolism**				
llmg_0154	***cbr***	carbonyl reductase	21.1	1.4e-13
llmg_0155^S^	***llmg_0155***	hypothetical protein	18.4	1.3e-12
llmg_0156^M^	*dltE*	oxidoreductase dltE	12.1	9.7e-9
**L. Replication, recombination and repair**				
llmg_0409	*ssbA*	single-stranded DNA-binding protein	4.3	8.4e-3
**O. Post-translational modification, protein turnover, and chaperones**				
llmg_0282	*nrdG*	anaerobic ribonucleoside-triphosphate reductase activating protein	-4.6	1.1e-4
**P. Inorganic ion transport and metabolism**				
llmg_1155	*llmg_1155*	Spx-like protein	9.9	3.5e-11
llmg_1138	*mtsA*	manganese ABC transporter substrate binding protein	4.6	3e-4
llmg_0335	***plpA***	D-methionine-binding lipoprotein plpA precursor	-5.7	6.1e-6
llmg_0336	***plpB***	D-methionine-binding lipoprotein plpB precursor	-4.9	3.6e-7
**R. General function prediction only**				
llmg_2172	*llmg_2172*	nitroreductase	6.9	3.1e-6
llmg_0095	***llmg_0095***	esterase	6.9	2.7e-7
llmg_0097	***llmg_0097***	flavoprotein oxygenase	4.9	9.3e-7
llmg_0087	*llmg_0087*	short-chain type dehydrogenase	5.3	9.6e-7
llmg_1115	*llmg_1115*	XpaC-like protein	4.3	3.6e-7
**S. Function unknown**				
llmg_2163^K^	***llmg_2163***	hypothetical protein	18.4	1.4e-13
llmg_2164	***llmg_2164***	hypothetical protein	18.4	2.3e-11
llmg_1659	*llmg_1659*	hypothetical protein	11.3	4.6e-14
llmg_1572	*mycA*	hypothetical protein	5.7	1.5e-8
llmg_0590	*llmg_0590*	hypothetical protein	4.9	2.1e-3
llmg_1263	*llmg_1263*	hypothetical protein	4.3	2.1e-6
llmg_1029	*llmg_1029*	hypothetical protein	4	8.2e-6
**T. Signal transduction mechanisms**				
llmg_1698	*llmg_1698*	hypothetical protein	4.9	4.6e-5
**V. Defense mechanisms**				
llmg_1675	***llmg_1675***	ABC transporter ATP-binding protein	9.6	1.9e-5
llmg_1676^M^	***llmg_1676***	ABC transporter permease	8.6	3.4e-4
**llmg_0328**^**X**^	*llmg_0328*	hypothetical protein	6.5	3e-4
**llmg_0329**	*llmg_0329*	ABC transporter ATP binding and permease	9.6	1.3e-4
**X. No predictions**				
llmg_0169	*llmg_0169*	hypothetical protein	16	3.6e-8
llmg_1200^E^	***llmg_1200***	hypothetical protein	4.6	2.4e-4
llmg_1201	***llmg_1201***	hypothetical protein	7.5	5.8e-6
**llmg_1210**^**G**^	*llmg_1210*	multidrug resistance protein	8	3.9e-11
**llmg_1211**	*llmg_1211*	hypothetical protein	6.5	1.1e-9
llmg_1283	*llmg_1283*	hypothetical protein	6.1	1.5e-4
llmg_0641	***llmg_0641***	hypothetical protein	5.3	1.6e-8
llmg_0643^P^	***pacL***	cation transporter E1-E2 family ATPase	8	5.9e-7
llmg_1198	*llmg_1198*	hypothetical protein	5.3	2.2e-3
llmg_0985	*llmg_0985*	hypothetical protein	4.9	6.6e-4
llmg_0710	*llmg_0710*	hypothetical protein	-4.6	3.3e-6

* ^E^ Amino acid transport and metabolism

^K^ Transcription

^G^ Carbohydrate transport and metabolism

^S^ Function unknown

^M^ Cell wall/membrane/envelope biogenesis

^P^ Inorganic ion transport and metabolism

^X^ No predictions

## Discussion

The capacity of bacterial strains to stabilize emulsions depends on the molecular composition of their cell walls and the resulting surface properties. During the screening of surface properties of 55 *L*. *lactis* strains it was shown that *L*. *lactis* cells can be dispersed in water but not in oil [29]. However, when cells with a high cell surface hydrophobicity (CSH) are mixed with a hydrocarbon, they disappear from the water phase and locate to the oil-water interphase of the emulsion formed. Importantly, the CSH and emulsion stability capacity are independent parameters [29]. We here observed that the hydrophobic *L*. *lactis* strains NCDO712, MG1363*pil*, MG1614_clu^+^ all form strong oil-in-water emulsions with petroleum but not with sunflower seed oil. Localisation of cells at the oil-water interphase has been observed previously upon emulsion formation with hydrocarbon [12]. For the first time this study shows that induced cell aggregation improves bacterial emulsification of a food-grade oil. The emulsion stability provided by the aggregated bacterial cells is, most probably, caused by hindering of the coalescence of oil droplets through Pickering stabilization.

If one considers bacterial cells in a food matrix as colloidal particles it is important to take into account that bacterial aggregates possesses a higher level of organization than other hydrocolloid particles such as proteins or polysaccharides. Another difference exists in the fact that the food (micro) environment not only changes outer surface properties of the cell [52], but also affects the cells’ responses [53] and as a consequence thereof their surfaces might change. Additionally, cell surface properties can vary with the growth phase of a cell [29]. These factors clearly distinguish bacteria from inert solid or colloidal particles as emulsion stabilizers.

We also describe the transcriptional response of microbial cells to residing at an oil-water interphase. A shift in the location of cells from a suspension to an oil-water interphase might alter their cellular metabolism and, in a food fermentation, possibly the production of certain flavour compounds. A profound response was evident in the transcriptome of bacteria incubated for 10 min after emulsion preparation. Especially genes involved in amino acid transport and metabolism were affected. Leucine, isoleucine, glutamate and histidine biosynthesis genes as well as dipeptide and oligopeptide transport genes were up-regulated when cells resided in the emulsion. Interestingly, all of the affected genes relating to amino acid metabolism are essential to *L*. *lactis* MG1363 [54], which is a plasmid-cured derivative of the strain NCDO712 used here. Most of the up-regulated amino acid metabolism-related genes are under control of the global transcriptional regulator CodY [55–57]. Highly likely, residing at an oil-water interphase is unfavourable for growth of lactococcal cells, either because nutrients such as amino acids become inaccessible upon localization at the oil droplet surface or because the amount of nutrients is very limited due to the high density of cells. The elevated expression of the histidine genes *hisC*,*Z*,*D*,*F*,*G* and the BCAA genes *leuABC* and *ilvBDH* suggests that *L*. *lactis* NCDO712 starts experiencing starvation as an earlier report showed evidence of high expression of these genes during starvation [58]. Genes for the transport of oligopeptides (the two *opp* operons), or dipeptides (*dppAP*, of which the former is functional, the latter is a pseudogene) and of branched-chain amino acids (*ctrA*, renamed *bcaP* [59]) are all under CodY control [59] and are all significantly up-regulated. This suggests an attempt of the cell to import peptides and/or amino acids as a response to the conditions of starvation. The up-regulated glutamate synthase GltB gene (gene *llmg_1183*) has been shown to be involved in acid stress response [60]. High cell densities at the oil-water interphase could potentially lead to high acid concentrations and induce the acid stress. In addition, a number of genes involved in citrate fermentation (*citB*, *icd*, *gltA*) were significantly up-regulated, which suggests that pH changes might also affect the expression of these genes. Citrate utilisation is strongly pH-dependent [61], however strain NCDO712 is not known to ferment citrate. Interestingly the *citB*, *icd*, *gltA* genes are also under control of CodY, as has been shown in MG1363 [62]. The fact that the transcriptomes of cells on an oil-water interphase and the control samples in suspension converge after 30 minutes suggests that the time points chosen for sampling RNA are well chosen to detect the specific transcriptional response to this environmental change. The transcriptome convergence after 30 minutes together with the high cell counts that can be recovered after incubation in emulsion also suggests that the conditions on the water-oil interphase are not too harsh for cell survival.

While there seems to be potential for the use of hydrophobic and/or aggregating LAB as clean-label emulsifiers, the amount of bacteria required using the current protocol would prohibit using them for bulk products. Therefore, the amount of cells needed to stabilize an emulsion would need to be reduced to make this a feasible approach. Our results also point out how in a complex environment such as a fermented dairy product a heterogeneous cellular response can be brought about by the location of an organism in a particular part of the food matrix. The possibility of selecting starter cultures with altered surface properties was demonstrated recently by conjugating a plasmid from *L*. *lactis* NCDO712 to a recipient strain that became lactose positive and showed a clumping phenotype (MG1614_clu^+^) [40]. Such approaches could be used to steer cells towards an oil-water interphase in a fermented product. This would change its direct environment and, potentially, its metabolic activity. It has previously been shown that flavour profiles could be changed by varying the size of microcolonies in a cheese matrix [51]. The authors speculate that this is due to the localized high cell densities in the colonies leading to altered metabolic activities [51].

Besides direct microbe-matrix interactions there is also an increasing interest in strain selection and screening protocols [49,63] employing microdroplets of oil [49] or alginate beads in which cells are cultured at high cell densities [64]. From the results presented here it is clear that when working with such systems it is important to understand how bacterial cell surface properties might influence the location of a cell within a droplet and how the resulting high cell densities could alter microbial metabolism.

## Materials and methods

### Bacterial strains, growth conditions and medium

Bacterial strains and plasmids are presented in Table A in S1 Tables. *L*. *lactis* strains were grown at 30°C in M17 (Oxoid, Thermo Scientific, Basingstoke, Hampshire, UK) supplemented with 1% lactose (LM17). When required, rifampicin (Rif; 50 μg/ml), streptomycin (Str; 100 μg/ml) or erythromycin (Ery; 10 μg/ml) was added to the indicated end-concentrations.

### Aggregation measurements

Cell from overnight cultures were harvested by centrifuging at 6037 x *g* for 3 min in 50 ml tubes, washed twice with phosphate buffer (10 mM, pH 6.8), and finally diluted to an optical density at 600 nm (OD_600_) of 1.0 in the same buffer.

The cell suspensions (1.5 ml each) were transferred to 2 ml Eppendorf tubes, centrifuged at 15339 x *g* for 30 sec, after which the supernatant was removed and the cell pellets were re-suspended in phosphate buffer containing 5–25% (v/v) ethanol. Subsequently, the OD_600_ was measured every 10 min for 1 h. The same approach was used to prepare bacterial samples in 10 mM phosphate buffer with 0.1–3.0 M ammonium sulfate. Cell aggregation was determined using Eq 1:

$$Cell\;aggregation\;(\%)=100\times \frac{{OD}_{600}\;at\;0\;\min-{OD}_{600}\;at\;1\;h}{{OD}_{600}\;at\;0\;min}.$$ (1)

### Emulsion preparation and cell surface hydrophobicity (CSH, %) measurement

Emulsions were prepared and cell surface hydrophobicity (CSH, %) was calculated as described previously [29]. The oil used was either petroleum (Sigma-Aldrich, Steinheim, Germany) or plant-derived oil (sunflower seed oil from *Helianthus annuus* (Sigma-Aldrich, #S5007-1L, Steinheim, Germany). For experiments with ethanol or ammonium sulfate either 5–25% ethanol or 0.1–3.0 M ammonium sulfate was added to the cell suspension prior to mixing with the oil. Five ml of the cell suspension in 10 mM phosphate buffer (with or without ethanol or ammonium sulfate) were mixed with 2 ml of the various oils including petroleum. The mixture was vortexed for 2 min and allowed to stand for 15 min for phase separation prior the measurements of OD at 600 nm.

### Confocal Laser Scanning Microscopy (CLSM)

Prior to the measurement cells from an overnight culture (10 ml of OD_600_ = 1) were spun down, washed twice with 10 mM phosphate buffer pH 6.8, and re-suspended in 100 μl of the same buffer. At room temperature and protected from light the cells were stained for 30 min, with Syto 60 (Thermo Fisher Scientific, Oregon, Hillsboro, USA) by adding 1 μl of the staining solution (5 mM in DMSO) to 1 ml of cell suspension (OD_600_ = 1). Buffer (10 mM phosphate) was separately prepared by adding carboxyfluorescein (200 ml buffer + 300 μl of 100 mM carboxyfluorescein (Sigma-Aldrich) stock solution in water) and kept out of the light.

After staining with Syto 60, the cell suspension was diluted until OD_600_ of 1 in the carboxyfluorescein buffer. This suspension (5 ml) was mixed with 2 ml petroleum, vortexed and allowed to stand for 20 min in the dark for proper phase separation. Then 300 μl of the emulsion (top layer) was transferred into a CLSM cuvette (NIZO, Ede, The Netherlands).

CLSM images were acquired with a Leica TCS SP5 confocal laser scanning microscope (Leica Microsystems CMS GmbH, Mannheim, Germany) with Leica application Suite Advanced Fluorescence v. 2.7.3. build. 9723. The Argon laser with excitation wavelength of 488 nm was used to visualize the carboxyfluorescein-stained buffer phase, while the HeNe633 laser with excitation wavelength of 633 nm was employed to visualize bacterial cells stained with Syto 60. The objective lens used was a Leica HCX PL APO 63×/1.2 /water CORR CS.

### Sample preparation for RNA sequencing

An overnight culture (27 ml, OD_600_ = 1.58) of *L*. *lactis* NCDO712 grown in chemically defined medium [65] with 1% lactose (LCDM) was diluted into 800 ml pre-warmed (30°C) fresh LCDM to an OD_600_ of 0.1 and distributed in 25-ml aliquots over 16 tubes of 50 ml. The cultures were incubated at 30°C until an OD_600_ of 0.43±0.03 was reached. Cells were harvested by centrifugation and re-suspended in 25 ml of fresh LCDM to an OD_600_ of 0.4. This cell suspension was mixed with 5 ml of the fluorinated oil HFE7500 (M3) and vortexed for 2 min in a Vortex Genie 2 vortexer (Scientific Industries, VWR International, Darmstadt, Germany) at maximum speed. The resulting oil-in-water emulsion was incubated at 30°C and samples (30 ml each) were taken after 0, 10, 20 and 30 min. As a control, cells were treated as above but the cell suspension did not contain HFE7500. All samples (30 ml each) were prepared in independent duplicates. The suspension and emulsion samples at time 0 min were immediately frozen in liquid nitrogen. Similarly, samples incubated for 10, 20 or 30 min were quick-frozen in liquid nitrogen. To break an emulsion, the sample was centrifuged at 2°C for 3 min at 6037 x *g*. The supernatant was removed and the cell pellet was re-suspended in 400 μl ice-cold Tris-EDTA-(TE)-buffer (pH 8). The cell suspension was transferred to a screw-cap tube containing 500 mg glass-beads (diameter of 75–150 μm). Freshly prepared extraction mixture (500 μL acidic phenol/chloroform (ratio 1:1), 30 μl 10% sodium dodecyl sulfate, and 30 μl 3M Na-acetate (pH 5.2)) was then added and the tube was frozen in liquid nitrogen and kept at -80°C before breaking the cells. Cells were broken in a Savant FastPrep FP120 “bead beater” (Thermo Savant, Illkirch, France) by beating three times for 40 s at a speed of 4.0 m/sec. The sample was cooled for 1 min on ice in between the steps. Subsequently, the suspension was centrifuged for 1 min at 4°C at 14000 x *g* in an Eppendorf centrifuge (Marshall Scientific, Hampton, NH, US). The supernatant (500 μl) was transferred to a fresh eppendorf tube, mixed with 400 μl cold (4°C) chloroform, and centrifuged for 1 min in an Eppendorf centrifuge at 14000 x *g* at 4°C to improve RNA yield. RNA was isolated with the High Pure RNA Isolation Kit (Roche Molecular Systems, Almere, The Netherlands) using the protocol of the manufacturer. RNA concentration was determined using a Nanodrop (Thermo Fisher Scientific, Wilmington, DE, US). RNA samples were sent for nucleotide sequencing (PrimBio Research Institute, Exton, USA) using an Ion Proton system using an Ion P1-chip (Life Technologies). Cell survival was determined by preparing an emulsion through mixing of 1 ml cells (OD~10 in L-CDM) with 1 ml of HFE7500 vigorously. The cell suspension as well as the emulsion were incubated for 30 minutes and dilutions were plated on M17 medium supplemented with 1% lactose. Colony forming units were counted after 1 day of incubation.

### Data analysis

Each sample had on average 9.1 ± 1.4 million reads. Raw gene expression data for the two biological replicates per sample were normalized for total counts per sample and analysed using EdgeR [66] with multiple testing corrected *p*-value using the false-discovery rate (method used: Benjamini & Hochberg [67]). Genes with a *p*-value below 0.01 and differential expression levels between emulsion and suspension higher than 4 fold were selected for further visualisation. Data visualization was done using R (https://cran.r-project.org/bin/windows/base/). The d3heatmap function using Euclidian distance matrices, average hierarchical clustering and data scaling was used to generate the heatmap.

## Supporting information

S1 Tables **Table A. Surface properties of the strains used in this study.** PCSH stands for cell surface hydrophobicity with petroleum (%), ST—stationary growth phase; EXP—exponential growth phase, E24 (%)—emulsion stability measured after 24 h in petroleum, ZP (mV)–charge. Number represents average ± standard deviation of three biological replications. **Table B. Numbers of significantly differentially expressed genes in different COG categories.** A gene is only represented when its expression level is 4-fold higher or lower (p < 0.01) in cells under the two conditions tested: 10 min of incubation at the oil-water interphase in an emulsion or in suspension.
(PDF)

S1 Table RNAseq data. List of all differentially expressed genes of *Lactococcus lactis* NCDO712.
(XLSX)

S1 Fig Effect of ammonium sulfate or ethanol on aggregation behavior of Lactococcus lactis NCDO712. Strain NCDO712 is mainly present in loose cells or diplococci (in PBS or in chemically defined medium) (top panels). The addition of either ammonium sulfate (AMS) or ethanol leads to the appearance of cell aggregates. The photos above were taken after 1–3 hours of incubation with AMS or ethanol. We noticed that longer incubation times lead to more aggregates.
(TIF)

S1 Movie Z-axis scan of an oil-in-water emulsions made with stationary *L. lactis* NCDO712 stained with Syto9. The water phase consists of 10 mM phosphate buffer stained with NileBlueA (10 μL of 0.5% solution in 1 ml buffer) and the oil phase consists of hexane (non-stained). The z-axis was scanned 74.8 μm deep into the emulsion with 22 steps of 3.4 μm.
(MP4)

S2 Movie Z-axis scan of an oil-in-water emulsions made with stationary *L. lactis* MG1614_lac+ (40) stained with Syto60. The water phase consists of 10 mM phosphate buffer stained with carboxyfluorescein and the oil phase consists of petroleum (non-stained). The z-axis was scanned 57.4 μm deep into the sample with 12 steps of 4.7 μm.
(MP4)

## References

[1] Katsikogianni M, Missirlis YF. Concise review of mechanisms of bacterial adhesion to biomaterials and of techniques used in estimating bacteria-material interactions. Eur Cell Mater. 2004;8: 37–57. Available: http://www.ncbi.nlm.nih.gov/pubmed/15593018 15593018 10.22203/ecm.v008a05

[2] An YH, Friedman RJ. Concise review of mechanisms of bacterial adhesion to biomaterial surfaces. J Biomed Mater Res. 1998;43: 338–48. 10.1002/(SICI)1097-4636(199823)43 9730073

[3] Navarre WW, Schneewind O. Surface proteins of Gram-positive bacteria and mechanisms of their targeting to the cell wall envelope. Microbiol Mol Biol Rev. 1999;63: 174–229. Available: http://www.pubmedcentral.nih.gov/articlerender.fcgi?artid=98962&tool=pmcentrez&rendertype=abstract 10066836 10.1128/mmbr.63.1.174-229.1999 PMC98962

[4] Donlan RM. Biofilms: microbial life on surfaces. Emerg Infect Dis. 2002;8: 881–90. 10.3201/eid0809.020063 12194761 PMC2732559

[5] Decho A. Microbial biofilms in intertidal systems: an overview. Cont Shelf Res. 2000;20: 1257–1273. Available: http://www.sciencedirect.com/science/article/pii/S0278434300000224

[6] Mandlik A, Swierczynski A, Das A, Ton-That H. Pili in Gram-positive bacteria: assembly, involvement in colonization and biofilm development. Trends in Microbiology. 2008 pp. 33–40. 10.1016/j.tim.2007.10.010 18083568 PMC2841691

[7] Garrett TR, Bhakoo M, Zhang Z. Bacterial adhesion and biofilms on surfaces. Prog Nat Sci. 2008;18: 1049–1056. 10.1016/j.pnsc.2008.04.001

[8] Atlas RM. Microbial hydrocarbon degradation—bioremediation of oil spills. J Chem Technol Biotecnhnology. 1991;52: 149–156.

[9] Das N, Chandran P. Microbial degradation of petroleum hydrocarbon contaminants: an overview. Biotechnol Res Int. 2011;2011: 1–13. 10.4061/2011/941810 21350672 PMC3042690

[10] Leroy F, De Vuyst L. Lactic acid bacteria as functional starter cultures for the food fermentation industry. Trends Food Sci Technol. 2004;15: 67–78. 10.1016/j.tifs.2003.09.004

[11] Tadros TF. Emulsion formation, stability, and rheology. Emuls Form Stab. 2013; 1–76. 10.1002/9783527647941.ch1

[12] Dorobantu LS, Yeung AKC, Foght JM, Gray MR. Stabilization of oil-water emulsions by hydrophobic bacteria. Appl Environ Microbiol. 2004;70: 6333–6336. 10.1128/AEM.70.10.6333-6336.2004 15466587 PMC522095

[13] Rosenberg M. Bacterial adherence to hydrocarbons: a useful technique for studying cell surface hydrophobicity. FEMS Microbiol Lett. 1984;22: 289–295. 10.1016/0378-1097(84)90026-0

[14] Dickinson E. Food emulsions and foams: stabilization by particles. Curr Opin Colloid Interface Sci. Elsevier B.V.; 2010;15: 40–49. 10.1016/j.cocis.2009.11.001

[15] van Loosdrecht MC, Lyklema J, Norde W, Schraa G, Zehnder AJ. The role of bacterial cell wall hydrophobicity in adhesion. Appl Environ Microbiol. 1987;53: 1893–7. Available: http://www.pubmedcentral.nih.gov/articlerender.fcgi?artid=204020&tool=pmcentrez&rendertype=abstract 2444158 10.1128/aem.53.8.1893-1897.1987 PMC204020

[16] Firoozmand H, Rousseau D. Microbial cells as colloidal particles: Pickering oil-in-water emulsions stabilized by bacteria and yeast. Food Res Int. Elsevier B.V.; 2016;81: 66–73. 10.1016/j.foodres.2015.10.018

[17] Xiao J, Li Y, Huang Q. Recent advances on food-grade particles stabilized Pickering emulsions: fabrication, characterization and research trends. Trends Food Sci Technol. Elsevier Ltd; 2016;55: 48–60. 10.1016/j.tifs.2016.05.010

[18] Azeredo J, Visser J, Oliveira R. Exopolymers in bacterial adhesion: interpretation in terms of DLVO and XDLVO theories. Colloids Surfaces B Biointerfaces. 1999;14: 141–148. 10.1016/S0927-7765(99)00031-4

[19] Poortinga AT, Bos R, Norde W, Busscher HJ. Electric double layer interactions in bacterial adhesion to surfaces. Surf Sci Rep. 2002;47: 1–32. 10.1016/S0167-5729(02)00032-8

[20] Rutter PR, Vincent B. Physicochemical interactions of the substratum, microorganisms, and the fluid phase In: Marshall KC, editor. Microbial Adhesion and Aggregation. Berlin, Heidelberg: Springer; 1984 pp. 21–38.

[21] Hermansson M. The DLVO theory in microbial adhesion. Colloids Surfaces B Biointerfaces. 1999;14: 105–119.

[22] Neu TR, Marshall KC. Bacterial polymers: physicochemical aspects of their interactions at interfaces. J Biomater Appl. 1990;5: 107–133. 10.1177/088532829000500203 2266486

[23] Bayoudh S, Othmane A, Mora L, Ben Ouada H. Assessing bacterial adhesion using DLVO and XDLVO theories and the jet impingement technique. Colloids Surfaces B Biointerfaces. 2009;73: 1–9. 10.1016/j.colsurfb.2009.04.030 19493661

[24] Ong YL, Razatos A, Georgiou G, Sharma MM. Adhesion forces between E. coli bacteria and biomaterial surfaces. Langmuir. 1999;15: 2719–2725. 10.1021/la981104e

[25] Delcour J, Ferain T, Deghorain M, Palumbo E, Hols P. The biosynthesis and functionality of the cell-wall of lactic acid bacteria. Antonie Van Leeuwenhoek. 1999;76: 159–84. Available: http://www.ncbi.nlm.nih.gov/pubmed/10532377 10532377

[26] Chapot-Chartier M-P, Kulakauskas S. Cell wall structure and function in lactic acid bacteria. Microb Cell Fact. BioMed Central Ltd; 2014;13: S9 10.1186/1475-2859-13-S1-S9 25186919 PMC4155827

[27] Boonaert CJP, Rouxhet PG. Surface of lactic acid bacteria: relationships between chemical composition and physicochemical properties. Appl Envir Microbiol. 2000;66: 2548 10.1128/AEM.66.6.2548-2554.2000.Updated PMC110580 10831437

[28] Ly MH, Vo NH, Le TM, Belin J-M, Waché Y. Diversity of the surface properties of *Lactococci* and consequences on adhesion to food components. Colloids Surf B Biointerfaces. 2006;52: 149–53. 10.1016/j.colsurfb.2006.04.015 16844359

[29] Tarazanova M, Huppertz T, Beerthuyzen M, van Schalkwijk S, Janssen P, Wels M, et al Cell surface properties of *Lactococcus lactis* reveal milk protein binding specifically evolved in dairy isolates. Front Microbiol. 2017;8:1691 10.3389/fmicb.2017.01691 28936202 PMC5594101

[30] Loosdrecht M, Lyklema J, Norde W, Schraa G, Zehnder AJB. Electrophoretic mobility and hydrophobicity as electrophoretic mobility and hydrophobicity as a measure to predict the initial steps of bacterial adhesion. Appl Environ Microbiol. 1987;53: 1898–1901. 3662520 10.1128/aem.53.8.1898-1901.1987 PMC204021

[31] Pelletier C, Bouley C, Cayuela C, Bouttier S, Bourlioux P, Bellon-Fontaine MN. Cell surface characteristics of *Lactobacillus casei* subsp. *casei*, *Lactobacillus paracasei* subsp. *paracasei*, and *Lactobacillus rhamnosus* strains. Appl Environ Microbiol. 1997;63: 1725–31. Available: http://www.pubmedcentral.nih.gov/articlerender.fcgi?artid=168469&tool=pmcentrez&rendertype=abstract 9143109 10.1128/aem.63.5.1725-1731.1997 PMC168469

[32] Rosenberg M, Gutnick D, Rosenberg E. Adherence of bacteria to hydrocarbons: a simple method for measuring cell-surface hydrophobicity. FEMS Microbiol Lett. Wiley Online Library; 1980;9: 29–33. Available: http://onlinelibrary.wiley.com/doi/10.1111/j.1574-6968.1980.tb05599.x/abstract

[33] Neu TR. Significance of bacterial surface-active compounds in interaction of bacteria with interfaces. Microbiol Rev. 1996;60: 151–66. Available: http://www.pubmedcentral.nih.gov/articlerender.fcgi?artid=239423&tool=pmcentrez&rendertype=abstract 8852899 10.1128/mr.60.1.151-166.1996 PMC239423

[34] Kankainen M, Paulin L, Tynkkynen S, von Ossowski I, Reunanen J, Partanen P, et al Comparative genomic analysis of *Lactobacillus rhamnosus* GG reveals pili containing a human-mucus binding protein. Proc Natl Acad Sci U S A. 2009;106: 17193–17198. 10.1073/pnas.0908876106 19805152 PMC2746127

[35] Heeres AS, Picone CSF, van der Wielen LAM, Cunha RL, Cuellar MC. Microbial advanced biofuels production: overcoming emulsification challenges for large-scale operation. Trends Biotechnol. Elsevier Ltd; 2014;32: 221–229. 10.1016/j.tibtech.2014.02.002 24630476

[36] Wongkongkatep P, Manopwisedjaroen K, Tiposoth P, Archakunakorn S, Pongtharangkul T, Suphantharika M, et al Bacteria interface Pickering emulsions stabilized by self-assembled bacteria-chitosan network. Langmuir. 2012;28: 5729–5736. 10.1021/la300660x 22443382

[37] Firoozmand H, Rousseau D. Tailoring the morphology and rheology of phase-separated biopolymer gels using microbial cells as structure modifiers. Food Hydrocoll. Elsevier Ltd; 2014;42: 204–214. 10.1016/j.foodhyd.2014.04.040

[38] Ly MH, Naïtali-Bouchez M, Meylheuc T, Bellon-Fontaine M-N, Le TM, Belin J-M, et al Importance of bacterial surface properties to control the stability of emulsions. Int J Food Microbiol. 2006;112: 26–34. 10.1016/j.ijfoodmicro.2006.05.022 16952409

[39] Ly MH, Aguedo M, Goudot S, Le ML, Cayot P, Teixeira JA, et al Interactions between bacterial surfaces and milk proteins, impact on food emulsions stability. Food Hydrocoll. 2008;22: 742–751. 10.1016/j.foodhyd.2007.03.001

[40] Tarazanova M, Huppertz T, Kok J, Bachmann H. Altering textural properties of fermented milk by using surface-engineered *Lactococcus lactis*. Microb Biotechnol. 2018;0: 1–11. 10.1111/1751-7915.13278 PMC6011991 29745037

[41] Burgain J, Scher J, Francius G, Borges F, Corgneau M, Revol-Junelles AM, et al Lactic acid bacteria in dairy food: Surface characterization and interactions with food matrix components. Adv Colloid Interface Sci. Elsevier B.V.; 2014;213: 21–35. 10.1016/j.cis.2014.09.005 25277266

[42] Burgain J, Scher J, Lebeer S, Vanderleyden J, Corgneau M, Guerin J, et al Impacts of pH-mediated EPS structure on probiotic bacterial pili–whey proteins interactions. Colloids Surfaces B Biointerfaces. Elsevier B.V.; 2015;134: 332–338. 10.1016/j.colsurfb.2015.06.068 26209966

[43] Jeanson S, Floury J, Gagnaire V, Lortal S, Thierry A. Bacterial colonies in solid media and foods: A review on their growth and interactions with the micro-environment. Front Microbiol. 2015;6:1284 10.3389/fmicb.2015.01284 26648910 PMC4664638

[44] van der Mei HC, van de Belt-Gritter B, Doyle RJ, Busscher HJ. Cell surface analysis and adhesion of chemically modified *streptococci*. J Colloid Interface Sci. 2001;241: 327–332. 10.1006/jcis.2001.7768

[45] Larsen N, Nissen P, Willats WGT. The effect of calcium ions on adhesion and competitive exclusion of *Lactobacillus* ssp. and *E*. *coli* O138. Int J Food Microbiol. 2007;114: 113–119. 10.1016/j.ijfoodmicro.2006.10.033 17234293

[46] Lin L, Rosenberg M, Taylor KG, Doyle RJ. Kinetic analysis of ammonium sulfate dependent aggregation of bacteria. Colloids Surfaces B Biointerfaces. 1995;5: 127–134. 10.1016/0927-7765(95)01189-P

[47] Millsap KW, Van Der Mei HC, Reid G, Busscher HJ. Physico-chemical and adhesive cell surface properties of *Lactobacillus* strains grown in old formula and new, standardized MRS medium. J Microbiol Methods. 1996;27: 239–242. 10.1016/S0167-7012(96)00937-2

[48] Rayner M, Marku D, Eriksson M, Sjöö M, Dejmek P, Wahlgren M. Biomass-based particles for the formulation of Pickering type emulsions in food and topical applications. Colloids Surfaces A Physicochem Eng Asp. Elsevier B.V.; 2014;458: 48–62. 10.1016/j.colsurfa.2014.03.053

[49] Bachmann H, Fischlechner M, Rabbers I, Barfa N, Branco dos Santos F, Molenaar D, et al Availability of public goods shapes the evolution of competing metabolic strategies. Proc Natl Acad Sci U S A. 2013;110: 14302–7. 10.1073/pnas.1308523110 23940318 PMC3761572

[50] Tatusov RL, Fedorova ND, Jackson JD, Jacobs AR, Kiryutin B, Koonin E V., et al The COG database: An updated vesion includes eukaryotes. BMC Bioinformatics. 2003;4 10.1186/1471-2105-4-4 12969510 PMC222959

[51] Ly-Chatain MH, Linh M, Le ML, Belin J, Waché Y, Thanh M Le, et al Cell surface properties affect colonisation of raw milk by lactic acid bacteria at the microstructure level. Food Res Int. Elsevier Ltd; 2010;43: 1594–1602. 10.1016/j.foodres.2010.04.019

[52] Kaczorek E, Moszyńska S, Olszanowski A. Modification of cell surface properties of *Pseudomonas alcaligenes* S22 during hydrocarbon biodegradation. Biodegradation. 2011;22: 359–66. 10.1007/s10532-010-9406-4 20820883 PMC3046353

[53] Yvon M, Gitton C, Chambellon E, Bergot G, Monnet V. The initial efficiency of the proteolytic system of *Lactococcus lactis* strains determines their responses to a cheese environment. Int Dairy J. 2011;21: 335–345. 10.1016/j.idairyj.2010.11.010

[54] Jensen PR, Hammer K. Minimal requirements for exponential-growth of *Lactococcus lactis*. Appl Environ Microbiol. 1993;59: 4363–4366. 10.1093/emboj/16.12.3533 16349136 PMC195913

[55] Guédon E, Sperandio B, Pons N, Ehrlich SD, Renault P. Overall control of nitrogen metabolism in *Lactococcus lactis* by CodY, and possible models for CodY regulation in *Firmicutes*. Microbiology. 2005;151: 3895–3909. 10.1099/mic.0.28186-0 16339935

[56] Hengst CD den, Buist G, Nauta A, Sinderen D Van, Kuipers OP, Kok J. Probing direct interactions between CodY and the *oppD* promoter of *Lactococcus lactis*. Microbiology. 2005;187: 512–521. 10.1128/JB.187.2.512 PMC543541 15629923

[57] Chambellon E, Yvon M. CodY-regulated aminotransferases AraT and BcaT play a major role in the growth of *Lactococcus lactis* in milk by regulating the intracellular pool of amino acids. Appl Environ Microbiol. 2003;69: 3061–3068. 10.1128/AEM.69.6.3061-3068.2003 12788699 PMC161493

[58] Ercan O, Wels M, Smid EJ, Kleerebezem M. Genome-wide transcriptional responses to carbon starvation in nongrowing *Lactococcus lactis*. Appl Environ Microbiol. 2015;81: 2554–2561. 10.1128/AEM.03748-14 25636846 PMC4357953

[59] den Hengst CD, Groeneveld M, Kuipers OP, Kok J. Identification and functional characterization of the *Lactococcus lactis* acid permease BcaP (CtrA). J Bacteriol. 2006;188: 3280–3289. 10.1128/JB.188.9.3280-3289.2006 16621821 PMC1447443

[60] Feehily C, Karatzas KAG. Role of glutamate metabolism in bacterial responses towards acid and other stresses. J Appl Microbiol. 2013;114: 11–24. 10.1111/j.1365-2672.2012.05434.x 22924898

[61] Starrenburg MJC, Hugenholtz J. Citrate fermentation by *Lactococcus* and *Leuconostoc* spp. Appl Environ Microbiol. 1991;57: 3535–3540. 16348602 10.1128/aem.57.12.3535-3540.1991 PMC184008

[62] Hengst CD Den Hijum SAFT Van, Geurts JMW, Nauta A, Kok J, Kuipers OP. The *Lactococcus lactis* CodY regulon. Identification of a conserved cis-regulatory element. J Biol Chem. 2005;280: 34332–34342. 10.1074/jbc.M502349200 16040604

[63] Chen J, Vestergaard M, Jensen TG, Shen J, Dufva M, Solem C, et al Finding the needle in the haystack-the use of microfluidic droplet technology to identify vitamin-secreting lactic acid bacteria. MBio. 2017;8: 1–12. 10.1128/mBio.00526-17 28559484 PMC5449655

[64] Le-Tien C, Millette M, Mateescu M-A, Lacroix M. Modified alginate and chitosan for lactic acid bacteria immobilization. Biotechnol Appl Biochem. 2004;39: 347–54. 10.1042/BA20030158 15154848

[65] Price CE, Branco dos Santos F, Hesseling A, Uusitalo JJ, Bachmann H, Benavente V, et al Adaption to glucose limitation is modulated by the pleotropic regulator CcpA, independent of selection pressure strength. BMC Evol Biol. 2019;19: 15 10.1186/s12862-018-1331-x 30630406 PMC6327505

[66] Robinson MD, Oshlack A. A scaling normalization method for differential expression analysis of RNA-seq data. Genome Biol. 2010;11: R25 10.1186/gb-2010-11-3-r25 20196867 PMC2864565

[67] Benjamini Y, Hochberg Y. Controlling the false discovery rate: a practical and powerful approach to multiple testing. Journal of the royal statistical society Series B (Methodological). 199557: 289–300. 10.2307/2346101

