# Supplementary material for: Transcriptional response of Lactococcus lactis during bacterial emulsification
Source: PLoS One. 2019 Jul 25;14(7):e0220048. doi: 10.1371/journal.pone.0220048 (PMC6657864; doi:10.1371/journal.pone.0220048)
Supplement: S1 Tables — Table A. Surface properties of the strains used in this study. PCSH stands for cell surface hydrophobicity with petroleum (%), ST—stationary growth phase; EXP—exponential growth phase, E24 (%)—emulsion stability measured after 24 h in petroleum, ZP (mV)–charge. Number represents average ± standard deviation of three biological replications. Table B. Numbers of significantly differentially expressed genes in different COG categories. A gene is only represented when its expression level is 4-fold higher or lower (p < 0.01) in cells under the two conditions tested: 10 min of incubation at the oil-water interphase in an emulsion or in suspension. (PDF) [file pone.0220048.s001.pdf]

S1 Tables

Transcriptional response of *Lactococcus lactis* during bacterial emulsification

Authors: M. Tarazanova[1,2,3], T. Huppertz [1,2,#], M. Starrenburg[1,2,3], T. Todt[4,5], S. van Hijum[1,4], J. Kok[2,3], H. Bachmann[1,2, *]

[1] NIZO, Ede, The Netherlands

[2] TI Food and Nutrition, Wageningen, The Netherlands

[3] Molecular Genetics, University of Groningen, Groningen, The Netherlands

[4] Radboud University Medical Centre CMBI, Nijmegen, The Netherlands

[5] HAN, University of Applied Sciences, Nijmegen, The Netherlands

*Corresponding author:

E-mail: Herwig.Bachmann@nizo.com (HB)

[#]Current address: FrieslandCampina, Amersfoort, The Netherlands

Running title: "*L. lactis* emulsification and transcriptional response"

Table A.

L. lactis strain	Characteristic	Auto-aggregation	Stationary growth phase			Exponential growth phase			Reference
			CSH, %	E24, %	ZP, mV	CSH, %	E24, %	ZP, mV	
MG1363	Plasmid-cured derivative of *L. lactis* NCDO712	no	6±0	0±0	-30±1	7±3	0±0	-39±2	(29)
MG1363*pil*	Ery^R; derivative of MG1363 harbouring pSH74 pilin operon	yes	92±1	85±15	-16±1	96±2	91±14	-13±3	#
NCDO712	*L. lactis* dairy isolate, contains the following plasmids: pLP712, pSH71, pSH72, pSH73, pSH74, pNZ712	yes	99±1	100±0	-21±2	99±1	49±3	-20±1	(29)
MG1614_clu$^+$	Rif^R, Str^R; Transconjugant, clumping phenotype, derivative of MG1614 harbouring pLP712 from strain NCDO712	yes	90±4	31±4	-36±0	94±1	0±0	-26±0	(40)

[#] Tarazanova M, Beerthuyzen M, Siezen R, Fernandez-Gutierrez MM, de Jong A, van der Meulen S, Kok J, Bachmann H. 2016. Plasmid complement of *Lactococcus lactis* NCDO712 reveals a novel pilus gene cluster. PLoS One 11:e0167970

Table B.

COG function category	No. of genes affected	
	Up-regulation	Down-regulation
C. Energy production and conversion	3	0
E. Amino acid transport and metabolism	26	2
G. Carbohydrate transport and metabolism	6	0
H. Coenzyme transport and metabolism	1	0
I. Lipid transport and metabolism	1	0
K. Transcription	2	0
L. Replication, recombination and repair	1	0
M. Cell wall/membrane/envelope biogenesis	2	0
O. Post-translational modification, protein turnover, and chaperones	0	1
P. Inorganic ion transport and metabolism	8	2
Q. Secondary metabolites biosynthesis, transport, and catabolism	1	0
R. General function prediction only	6	0
S. Function unknown	7	0
T. Signal transduction mechanisms	2	0
V. Defense mechanisms	2	0
X. No predictions	8	1
Total [a]	76	6

[a] Some genes are assigned to more than 1 COG category
