# Supplementary figures and images for: Transcriptional response of Lactococcus lactis during bacterial emulsification
Source: PLoS One. 2019 Jul 25;14(7):e0220048. doi: 10.1371/journal.pone.0220048 (PMC6657864; doi:10.1371/journal.pone.0220048)

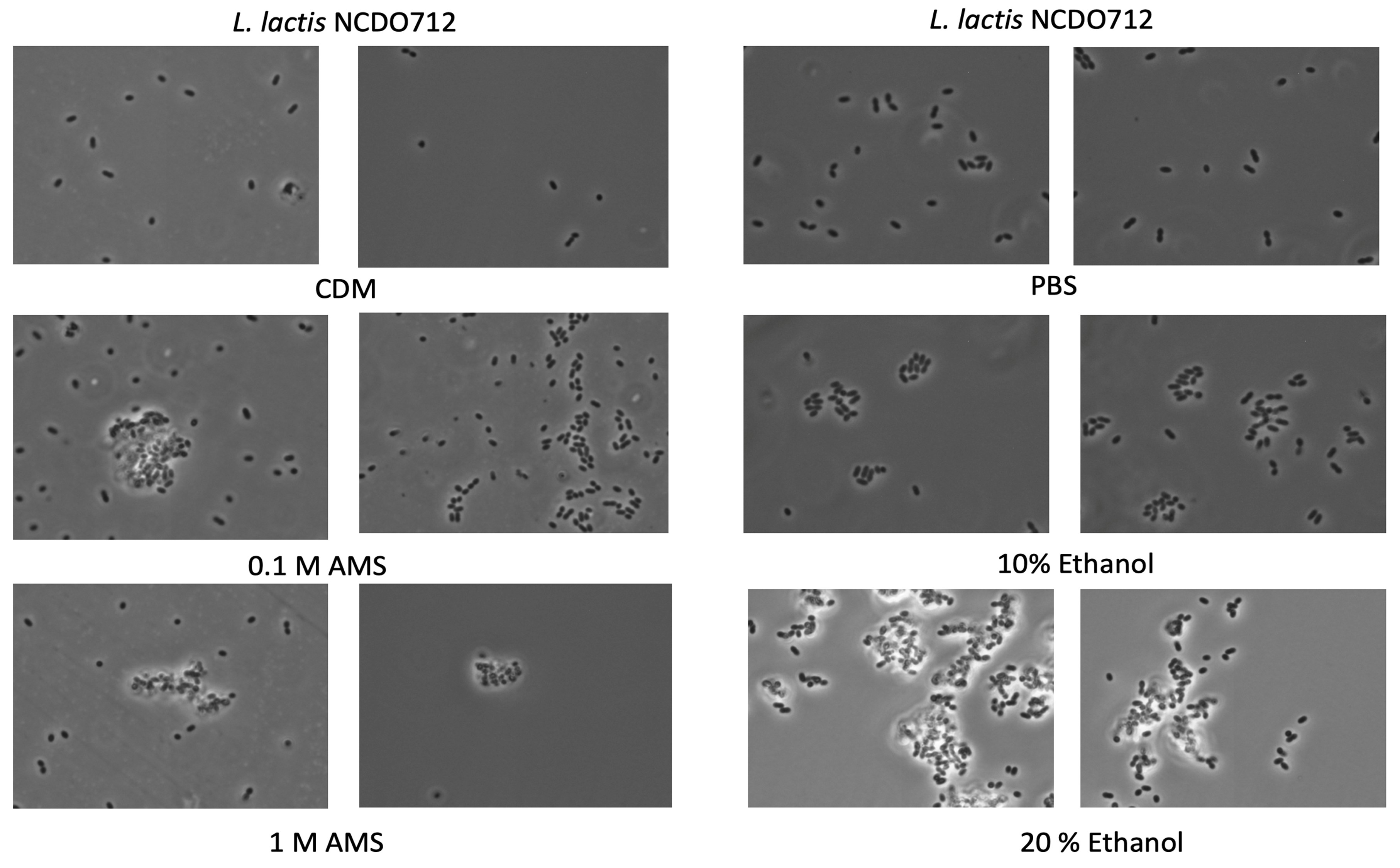

Supplement: S1 Fig — Strain NCDO712 is mainly present in loose cells or diplococci (in PBS or in chemically defined medium) (top panels). The addition of either ammonium sulfate (AMS) or ethanol leads to the appearance of cell aggregates. The photos above were taken after 1–3 hours of incubation with AMS or ethanol. We noticed that longer incubation times lead to more aggregates. (TIF) [file pone.0220048.s003.tif]
